# Refractory Acute Myeloid Leukemia following Extended Peptide Receptor Radionuclide Therapy for Well-Differentiated GEP-NETs: A Case Report

**DOI:** 10.1159/cro/abzag046

**Published:** 2026-09-05

**Authors:** Alda Aleksi, Sarah Hussain, Michael Asamoah-Boaheng

**Affiliations:** a Department of Medical Oncology, Arthur J.E Child Comprehensive Cancer Center, Calgary, AB, Canada; b Cancer Care Alberta, Arthur J.E Child Comprehensive Cancer Center, Calgary, AB, Canada; c Arnie Charbonneau Cancer Institute, University of Calgary, Calgary, AB, Canada

**Keywords:** 177Lu-DOTATATE, Therapy-related acute myeloid leukemia, Gastroenteropancreatic neuroendocrine tumor, Hematologic toxicity

## Abstract

**Introduction:**

Peptide receptor radionuclide therapy (PRRT) using ^177^Lu-DOTATATE is an established treatment for somatostatin receptor-positive gastroenteropancreatic neuroendocrine tumors. However, the incidence and risk profile of therapy-related myeloid neoplasms following extended ^177^Lu-DOTATATE exposure particularly beyond the standard four-cycle regimen remain poorly defined.

**Case Presentation:**

We report a 49-year-old woman with a well-differentiated jejunal neuroendocrine tumor with hepatic and peritoneal metastases who underwent bowel resection and limited hepatic metastatectomy, followed by long-acting somatostatin analog therapy and subsequent enrollment in a provincial PRRT clinical trial. She received 12 cycles of 177Lu-DOTATATE over 5 years (4 induction, 8 maintenance), without prior cytotoxic chemotherapy or external-beam radiotherapy, achieving durable biochemical and radiologic disease stability with minimal early toxicity. In late 2023, she developed progressive pancytopenia. Bone marrow examination in September 2024 revealed high-risk therapy-related acute myeloid leukemia (AML) characterized by inv(3)(q21q26.2), monosomy 7, complex cytogenetics, and a KRAS mutation. Her leukemia was refractory to intensive induction and subsequent lower-intensity therapy. She died on January 13, 2025, from progressive refractory AML with multiorgan dysfunction and central nervous system involvement.

**Conclusion:**

This case highlights a rare but life-threatening late hematologic toxicity associated with extended ^177^Lu-DOTATATE exposure and underscores the importance of long-term hematologic surveillance and critical evaluation of PRRT maintenance strategies beyond the standard four-cycle protocol.

## Introduction

Small-bowel neuroendocrine tumors (NETs) represent the most prevalent subtype of gastroenteropancreatic neuroendocrine tumors (GEP-NETs) [1]. Clinical outcomes are largely dictated by tumor grade, stage, proliferative index, mitotic activity, and primary site of origin. In patients with well-differentiated, grade 1–2 gastrointestinal NETs, median overall survival ranges from 55 to 65 months [2]. When feasible, surgical resection of the primary tumor and hepatic metastases remains the cornerstone of management; however, recurrence rates remain substantial [3, 4].

Somatostatin analogs (SSAs), including octreotide long-acting release and lanreotide, are routinely employed in patients with somatostatin receptor (SSTR)-positive NETs for symptom control and disease stabilization, particularly in the setting of functional tumors or high tumor burden. Peptide receptor radionuclide therapy (PRRT) represents a major therapeutic advance, delivering targeted β-emitting radiation via radiolabeled SSAs such as ^177^Lu-DOTATATE [5, 6]. The clinical efficacy of ^177^Lu-DOTATATE was firmly established in the NETTER-1 and NETTER-2 trials, which demonstrated significant improvements in progression-free survival (PFS) and objective response rates following 4 treatment cycles in patients with advanced GEP-NETs [7, 8].

Across prospective trials and large retrospective series, long-term hematologic toxicities – including myelodysplastic syndrome (MDS) and therapy-related myeloid neoplasms (TR-MNs) – have been reported in approximately 2–4% of patients, with acute myeloid leukemia (AML) occurring in fewer than 1% [9]. However, data addressing the safety profile of extended ^177^LU-DOTATATE exposure, particularly regimens exceeding the standard four treatment cycles, remain extremely limited.

In Alberta, Canada, access to PRRT has been available for nearly two decades prior to the approval of four cycles of ^177^Lu-DOTATATE as the current standard of care for unresectable metastatic GEP-NETs. This access has largely been facilitated through a provincial phase II clinical trial offering up to 12 cycles of ^177^Lu-octreotide therapy, consisting of 4 induction cycles followed by up to 8 maintenance cycles. Comprehensive long-term safety data associated with the administration of 12 PRRT cycles, including formal reporting of hematologic toxicity rates by this trial, are currently awaited. Here, we report a compelling case of high-risk therapy-related AML developing after 12 cycles of ^177^Lu-DOTATATE in a patient without prior exposure to cytotoxic chemotherapy.

## Trial Evidence Supporting PRRT Use in GEP-NETs

PRRT is a targeted therapeutic modality that delivers β-particle-emitting radionuclides to SSTR-expressing tumors through radiolabeled SSAs in patients with GEP-NETs [10]. PRRT with ^177^Lu-DOTATATE has become a well-established treatment for advanced GEP-NETs following the pivotal NETTER-1 and NETTER-2 phase III clinical trials.

NETTER-1 established four cycles of ^177^Lu-DOTATATE as an effective treatment for progressive midgut NETs, demonstrating substantial improvements in PFS and objective response rate compared with high-dose octreotide [7, 10]. NETTER-2 subsequently demonstrated improved PFS and response rates with first-line ^177^Lu-DOTATATE in patients with newly diagnosed grade 2–3 GEP-NETs [8]. Although hematologic toxicity was uncommon in both trials, long-term data indicate an estimated incidence of therapy-related MDS of 2–4% and acute leukemia of less than 1%. Reported risk factors include advanced age, bone metastases, extensive prior treatment, and exposure to alkylating agents such as temozolomide [11].

For nearly 2 decades before ^177^Lu-DOTATATE became the standard of care for unresectable metastatic GEP-NETs, access to PRRT in Alberta, Canada, was largely provided through a provincial phase II open-label clinical trial conducted at the Cross Cancer Institute in Edmonton. Until 2025, this trial represented the sole pathway for patients with advanced GEP-NETs in the province to receive ^177^Lu-DOTATATE following progression on SSAs. Enrollment required baseline functional assessment with a ^177^Lu-octreotide scan and complementary metabolic and anatomic imaging, with priority given to patients demonstrating favorable functional-imaging characteristics. The treatment protocol consisted of four induction cycles of ^177^Lu-DOTATATE administered at intervals of 8–12 weeks, followed by up to eight maintenance cycles administered every 6–9 months in patients who achieved radiologic stability after induction. Consequently, patients could receive up to 12 cycles of ^177^Lu-DOTATATE over 5 years.

A substantial proportion of patients treated through this protocol were referred from the Arthur J.E. Child Comprehensive Cancer Centre in Calgary, as the trial represented the only provincial PRRT option for many years. The present report describes a fatal case of PRRT-associated hematologic toxicity – specifically AML – following 12 cycles of ^177^Lu-DOTATATE in Alberta, Canada.

Available evidence from this trial currently consists of a conference abstract and a detailed master’s thesis from the University of Alberta reporting improvements in PFS and symptom control [12, 13]. The complete results of this longitudinal phase II study are awaited and are expected to provide valuable additional insights once published.

This case report was prepared and reported in accordance with the CARE (CAse REport) guidelines. The completed CARE checklist is provided as online supplementary material (for all online suppl. material, see https://doi.org/10.1159/cro/abzag046).

## Case Presentation

A 49-year-old woman with a past medical history notable only for migraines presented in 2017 with chronic right-sided abdominal and flank pain, progressive loose stools, and a newly palpable abdominal mass. Diagnostic laparoscopy revealed a large mesenteric mass originating from the proximal jejunum, encasing the superior mesenteric vessels, with concomitant multifocal hepatic and peritoneal metastases. Histopathologic evaluation of biopsy specimens confirmed a well-differentiated grade 1 small-bowel NET with a Ki-67 proliferation index of <2%. Baseline biochemical evaluation demonstrated mild elevations in 24-h urinary 5-hydroxyindoleacetic acid (51 mmol/day) and chromogranin A (177 ng/mL).

Following multidisciplinary tumor board review in 2018, the patient underwent surgical resection of approximately 60 cm of small bowel containing more than 30 tumor deposits, in addition to hepatic wedge resection (segment VII) for metastatic disease. The dominant mesenteric mass was deemed unresectable due to extensive encasement of the superior mesenteric artery and vein and was therefore left in situ. Postoperatively, the patient was initiated on octreotide long-acting release (Sandostatin LAR) at a dose of 30 mg administered every 4 weeks. She reported no postoperative symptoms suggestive of a functional hypersecretory syndrome, including diarrhea, bowel dysfunction, or flushing.

In 2018, radiologic disease progression was documented on cross-sectional imaging while receiving SSA therapy, prompting referral for PRRT. She subsequently underwent screening for enrollment in the provincial ^177^Lu-DOTATATE PRRT clinical trial, which required confirmation of progressive disease on anatomic imaging and positive SSTR expression on functional imaging.

Baseline abdominal MRI performed in June 2018 demonstrated the primary mesenteric mass on T1-weighted imaging (Fig. 1) and multiple hepatic lesions with restricted diffusion consistent with metastatic disease (Fig. 2). Faint peritoneal enhancement was also attributed to either postoperative changes or residual disease. Baseline dual functional imaging consisted of an octreotide scan and fluorodeoxyglucose positron emission tomography. The octreotide scan demonstrated intense radiotracer uptake within the mesenteric mass and mild uptake in a mesenteric lymph node, along with a nonspecific hepatic lesion raising suspicion for new liver metastases. Fluorodeoxyglucose positron emission tomography revealed a focal area of moderate hypermetabolism in the right hepatic lobe and a 3-cm mesenteric mass with low-grade uptake, corresponding to residual NET (Fig. 3–5, 6, and 7). Based on strong octreotide avidity and only moderate FDG uptake, she met eligibility criteria for trial enrollment.

**Fig. 1.**
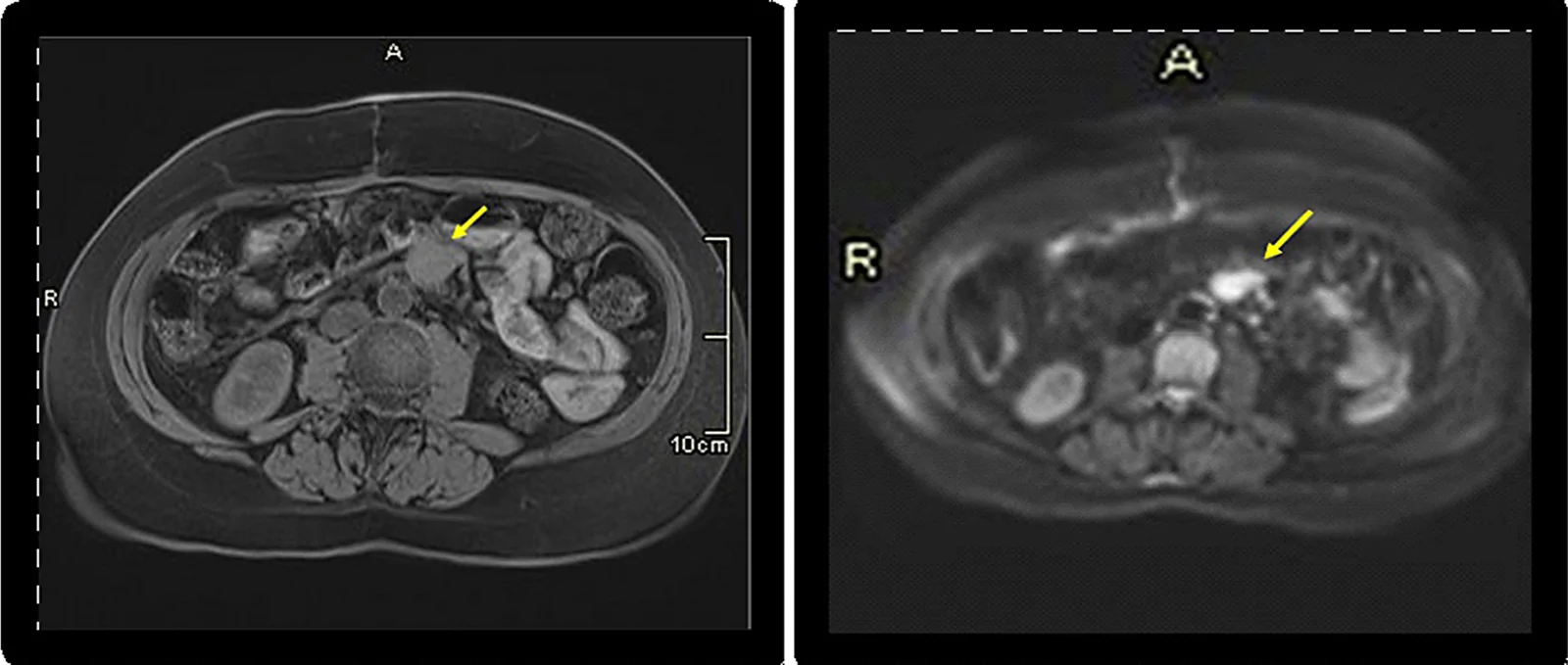
Baseline MRI, T1 mesenteric mass (arrows identify the primary mesenteric mass).

**Fig. 2.**
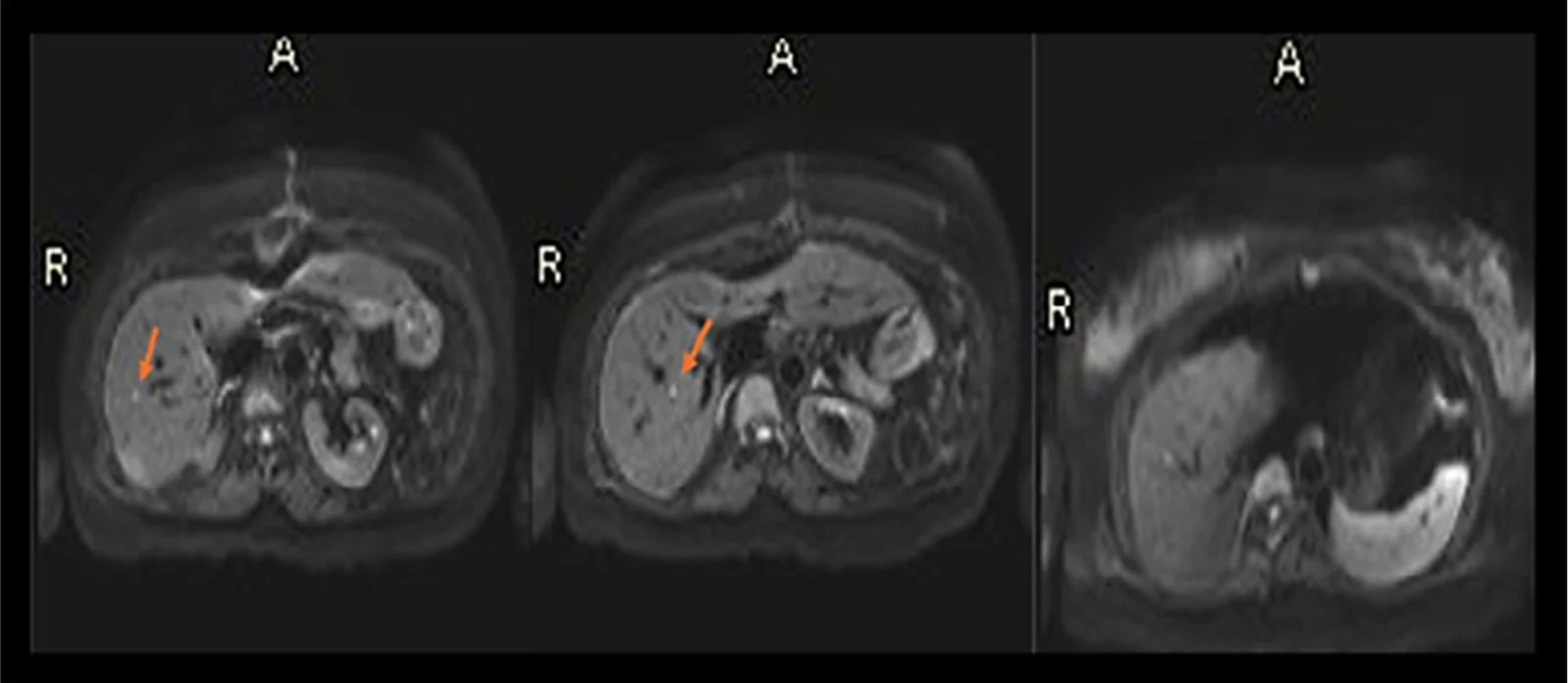
Baseline MRI, DWI (arrows identify liver lesions).

**Fig. 3.**
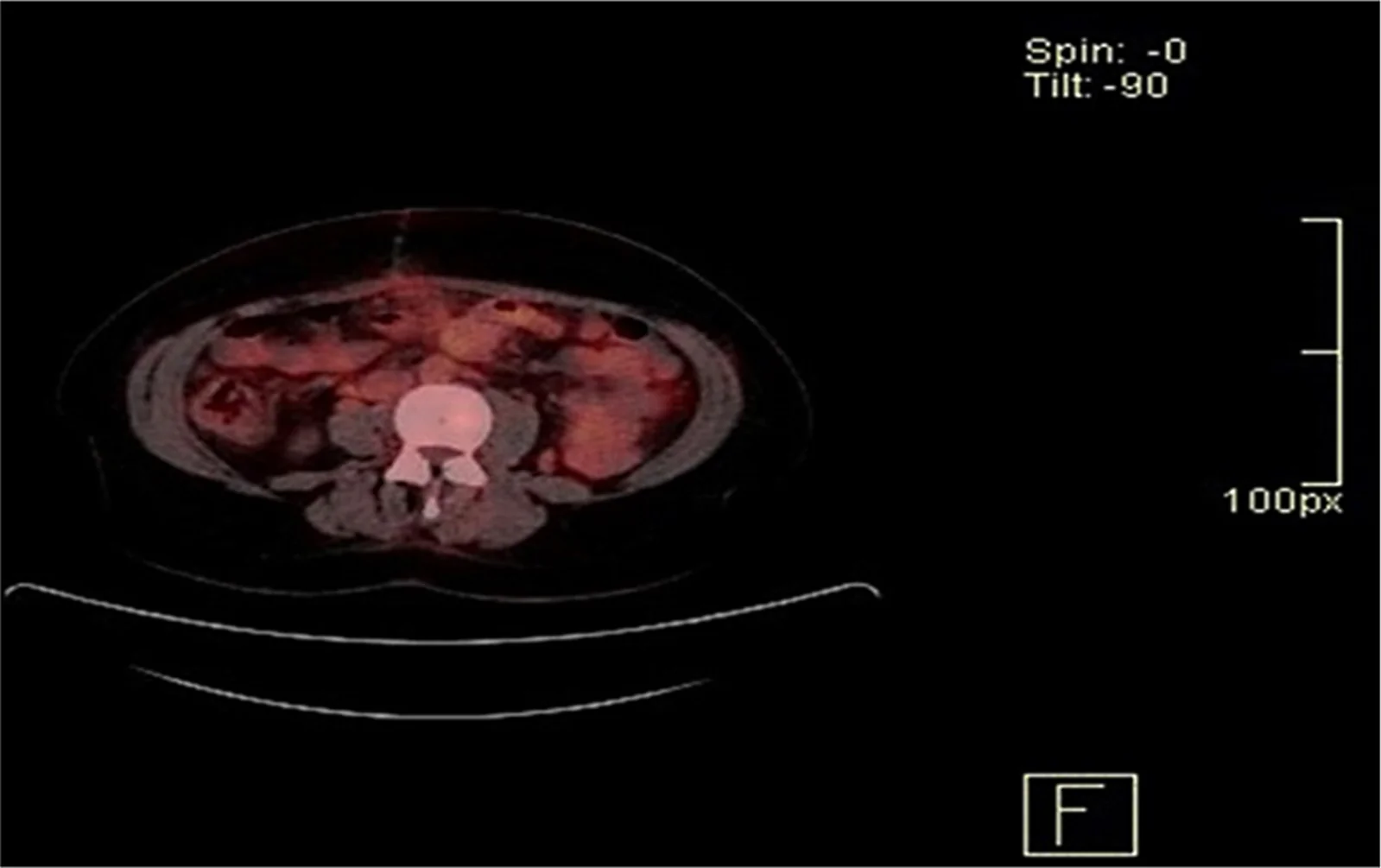
Baseline FDG-PET (mesenteric mass).

**Fig. 4.**
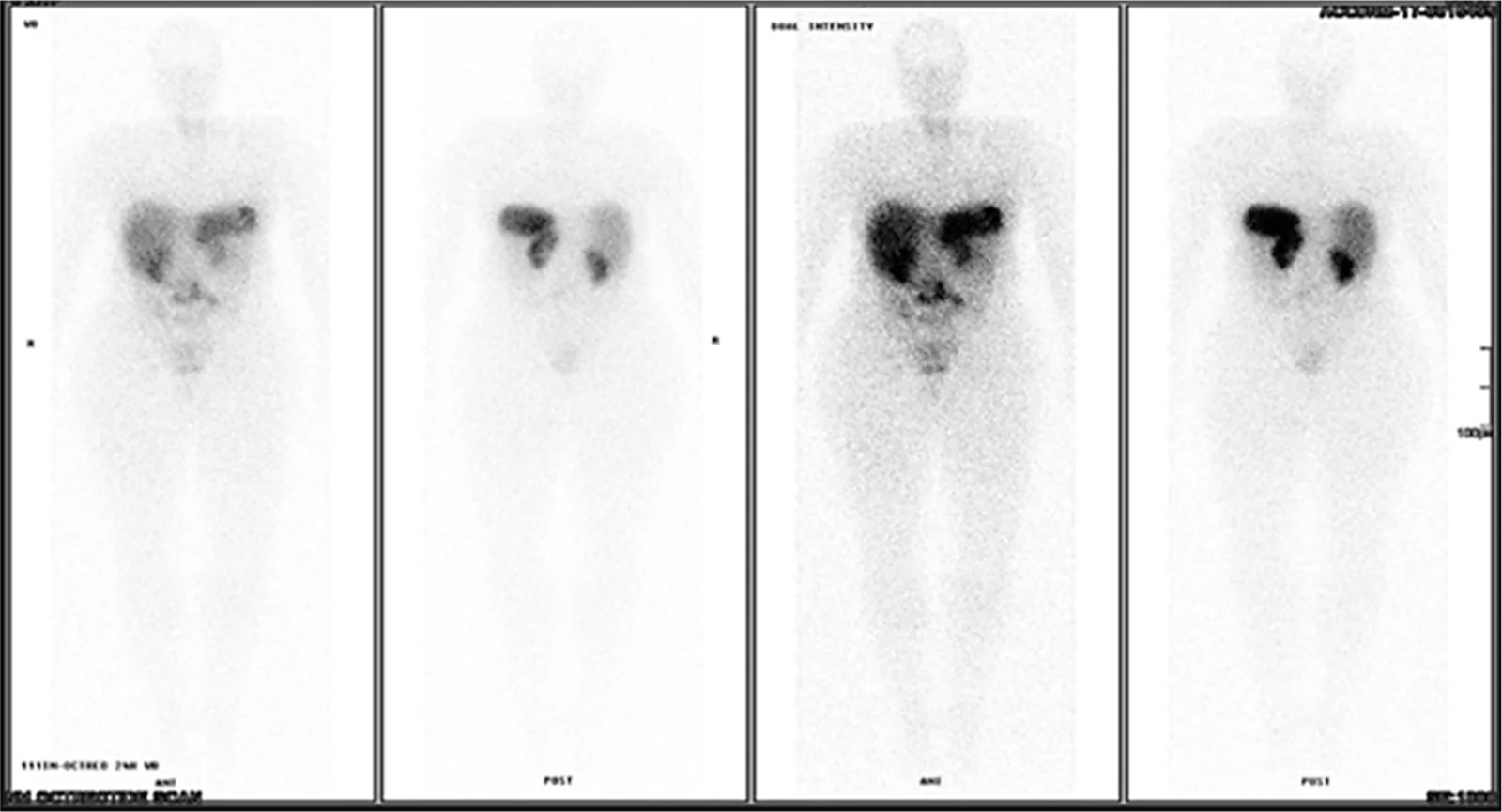
Baseline SSTR imaging with octreotide PET scan.

**Fig. 5.**
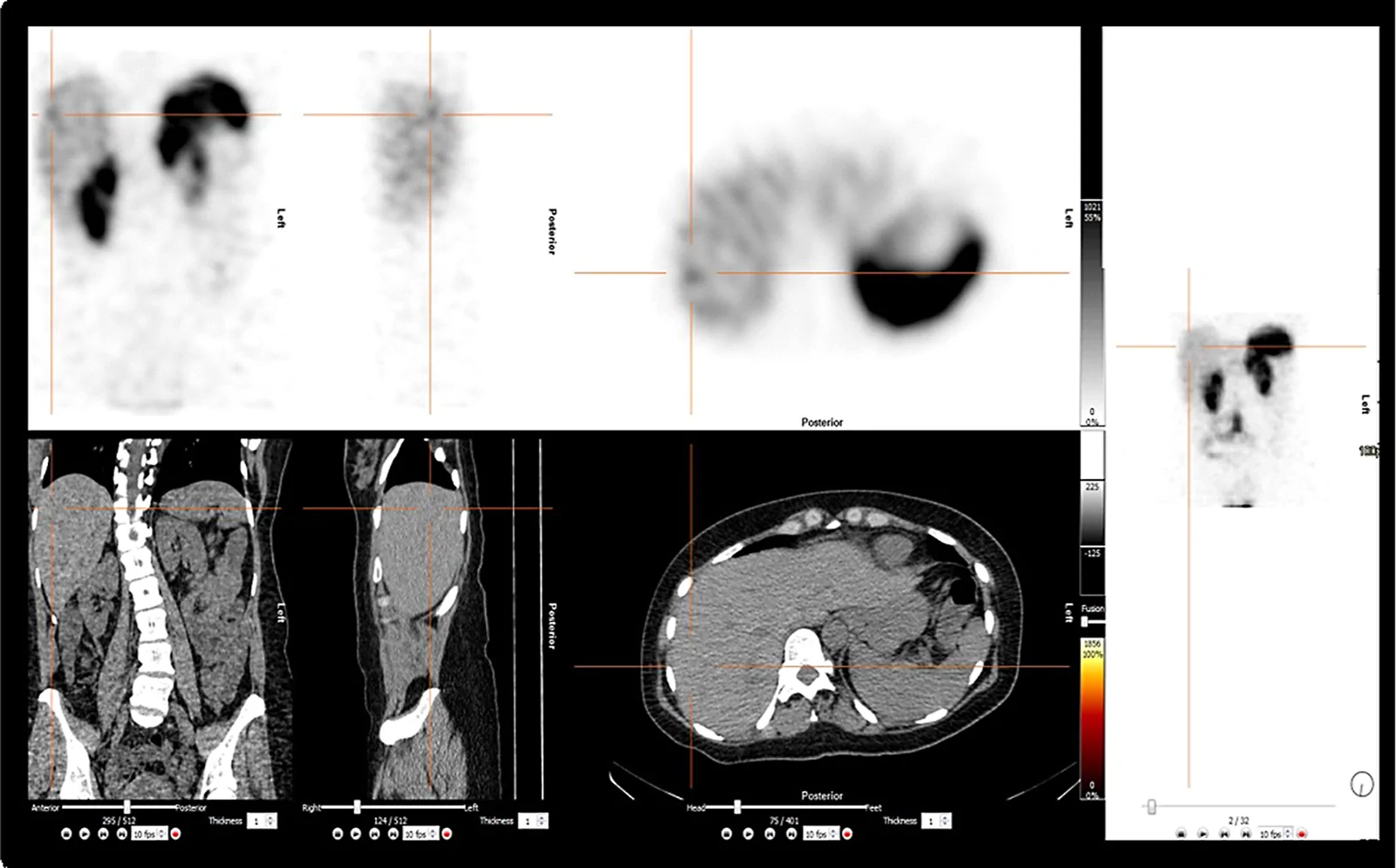
Baseline NM octreotide scan (xfused images 1).

**Fig. 6.**
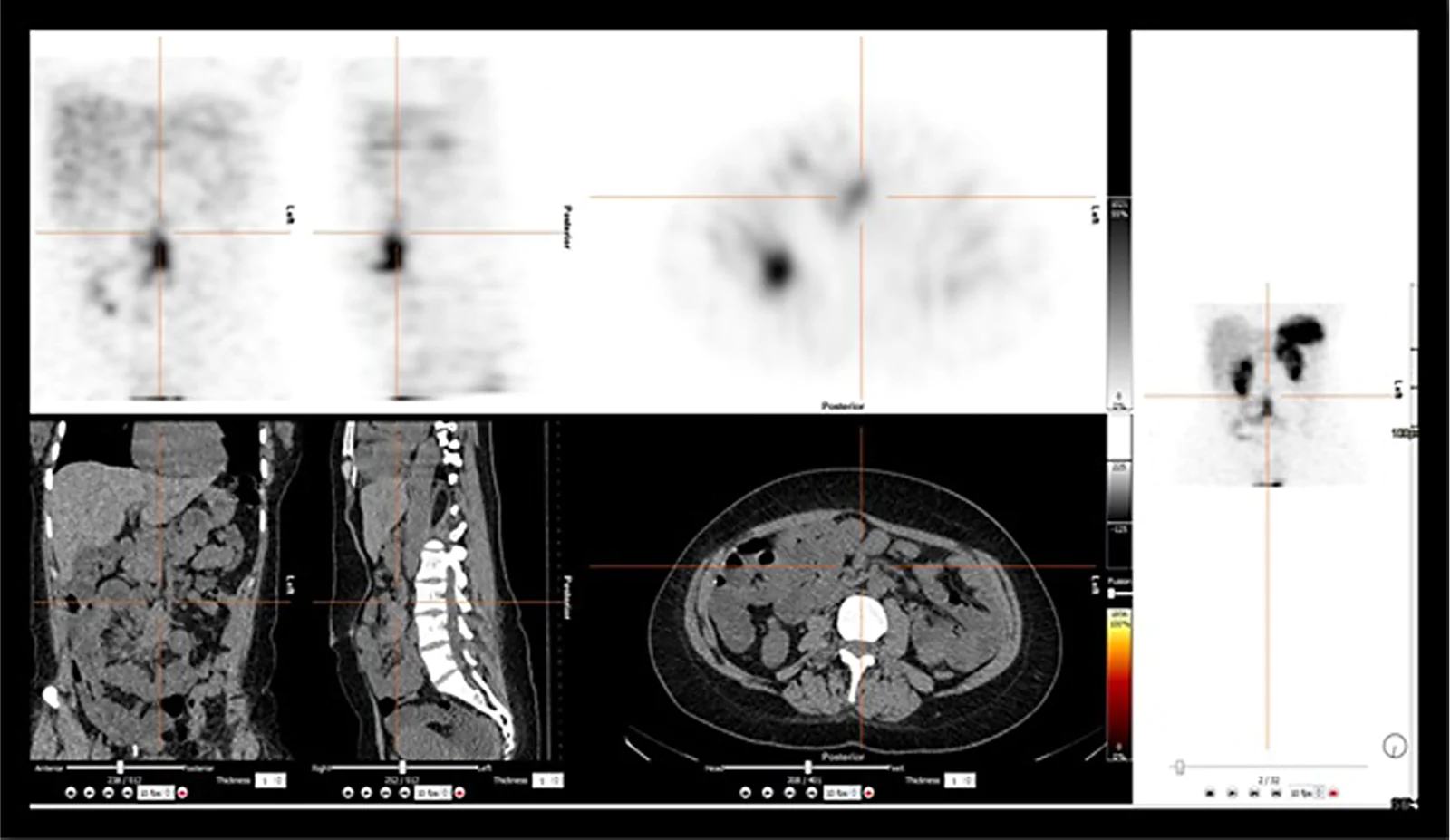
Baseline NM octreotide scan (fused images 2).

**Fig. 7.**
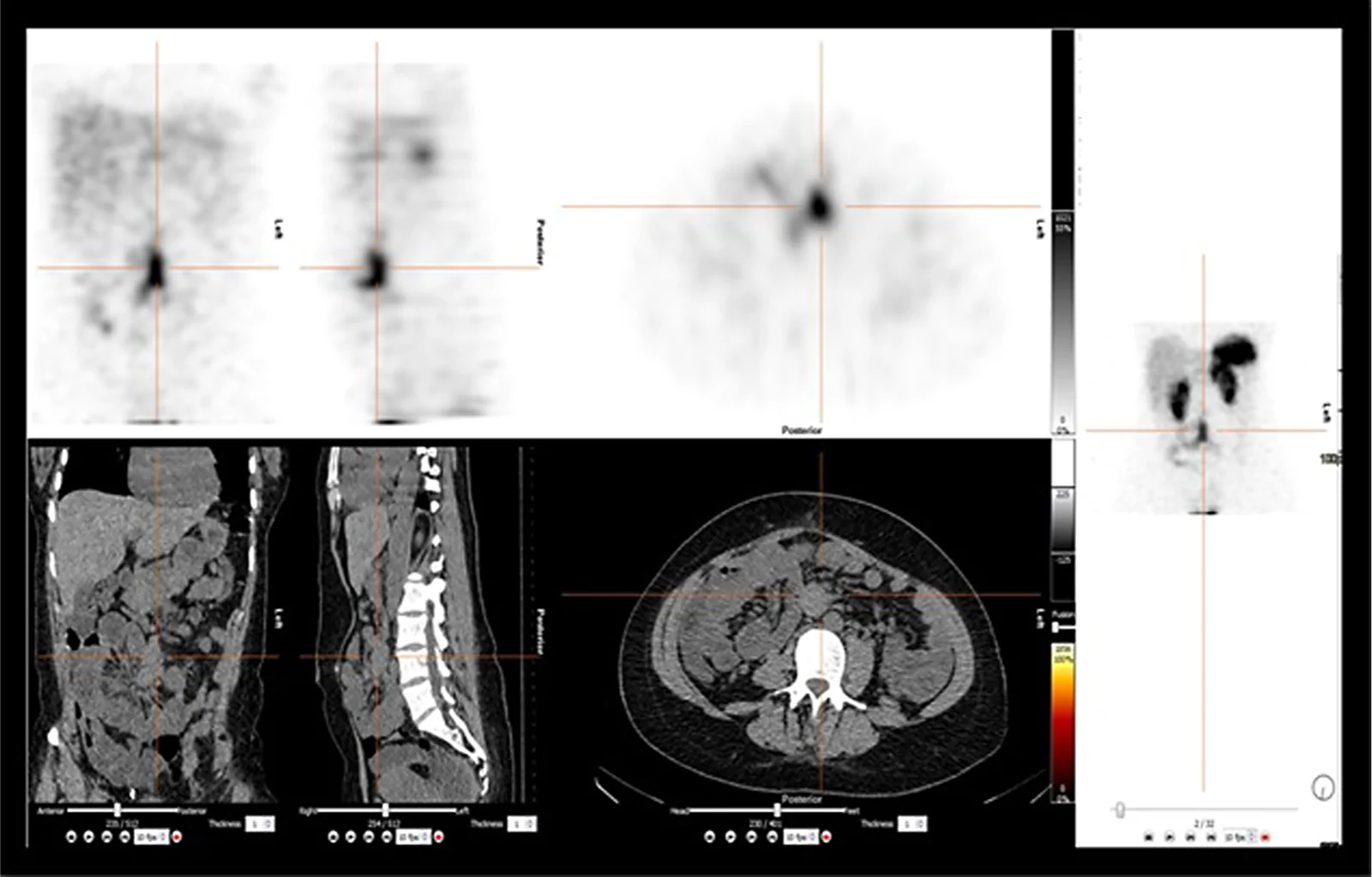
Baseline NM octreotide scan (fused images 3).

The patient was enrolled in the trial and received a total of 12 cycles of 177Lu-octreotide PRRT between September 2018 and November 2023. Post-treatment SPECT/CT imaging was performed following each cycle to confirm tumor uptake. Treatment was generally well tolerated, with only early grade 1–2 gastrointestinal toxicity. Following completion of four induction cycles, SPECT imaging demonstrated radiologic disease stability (Fig. 8, 9) with no change in the mesenteric mass (Fig. 10, 11), and maintenance PRRT was recommended per trial protocol. Biochemical markers improved substantially, with marked reductions in both 5-hydroxyindoleacetic acid and chromogranin A levels.

**Fig. 8.**
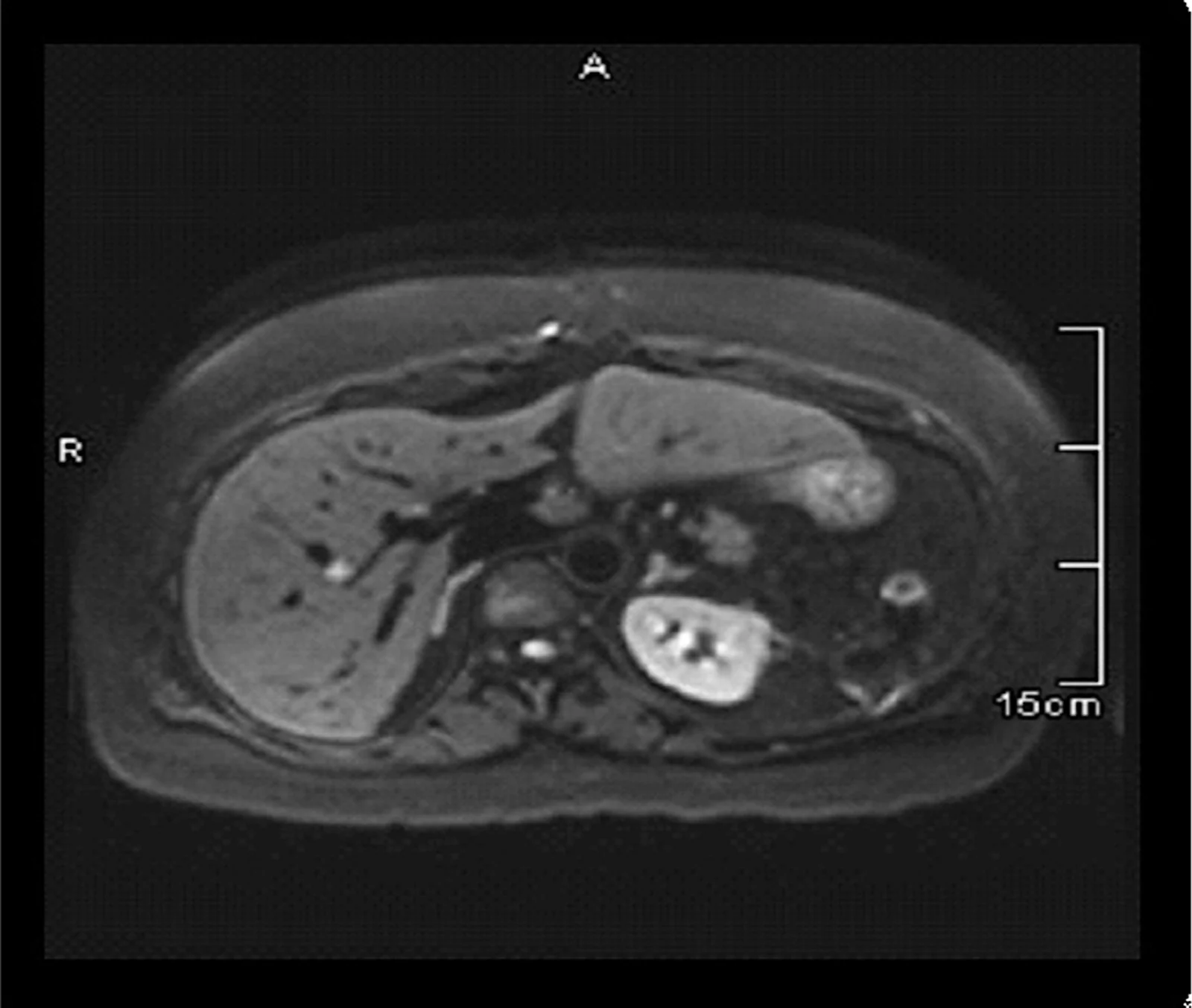
MRI, DWI (post 4# of PRRT).

**Fig. 9.**
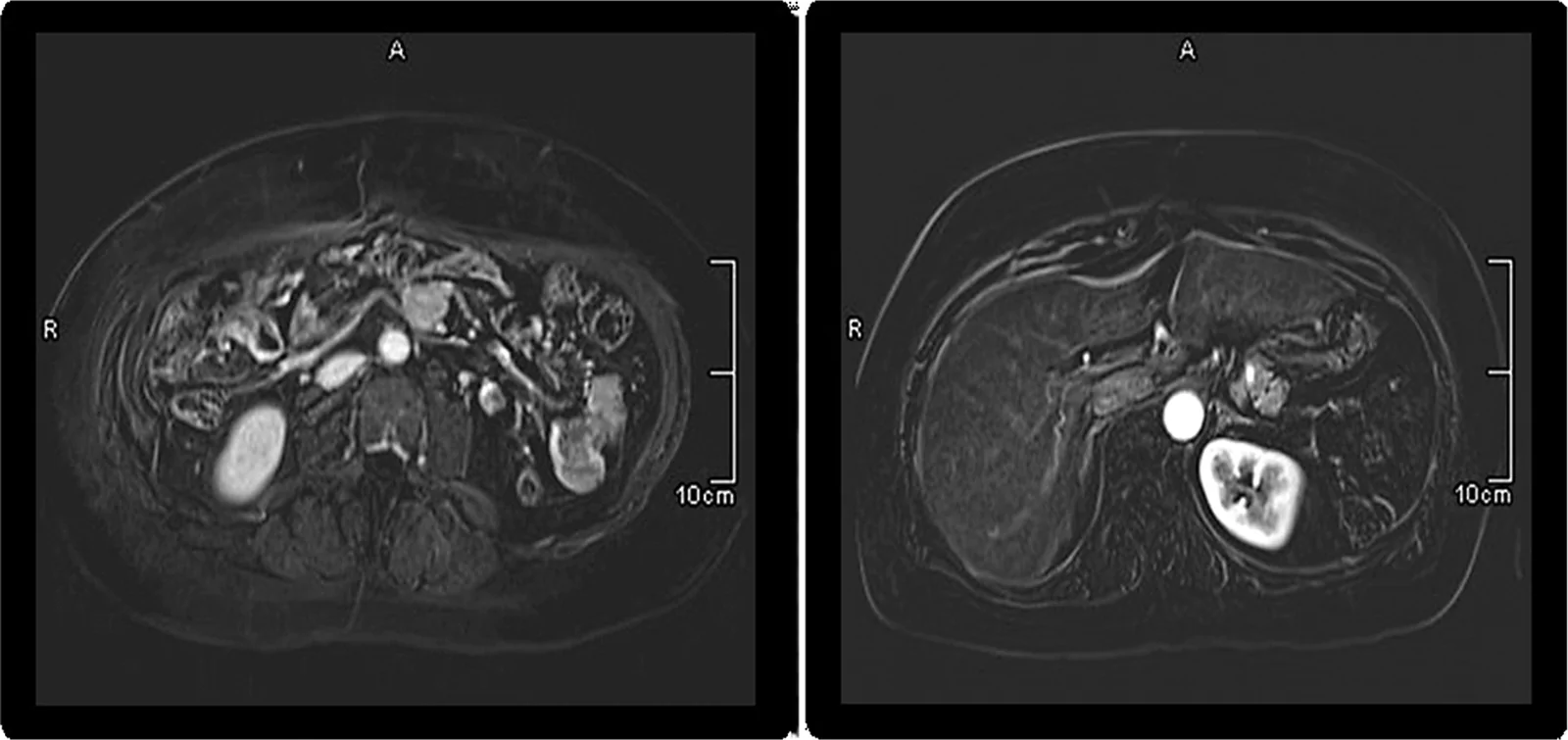
MRI, T1 gadolinium enhancement of mesenteric mass and liver lesion (post 4# of PRRT).

**Fig. 10.**
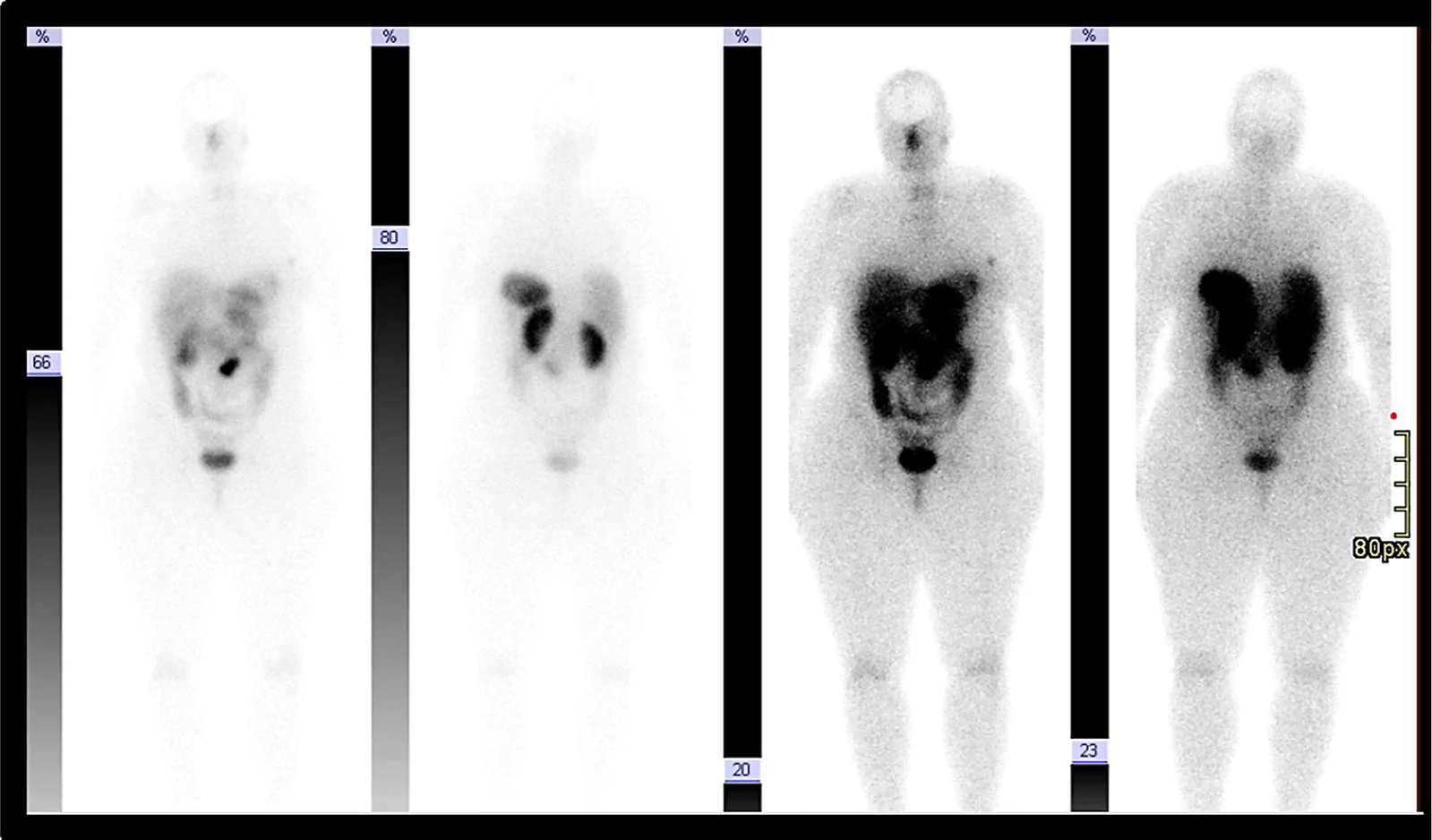
NM, octreotide SPECT (post 4# of PRRT).

**Fig. 11.**
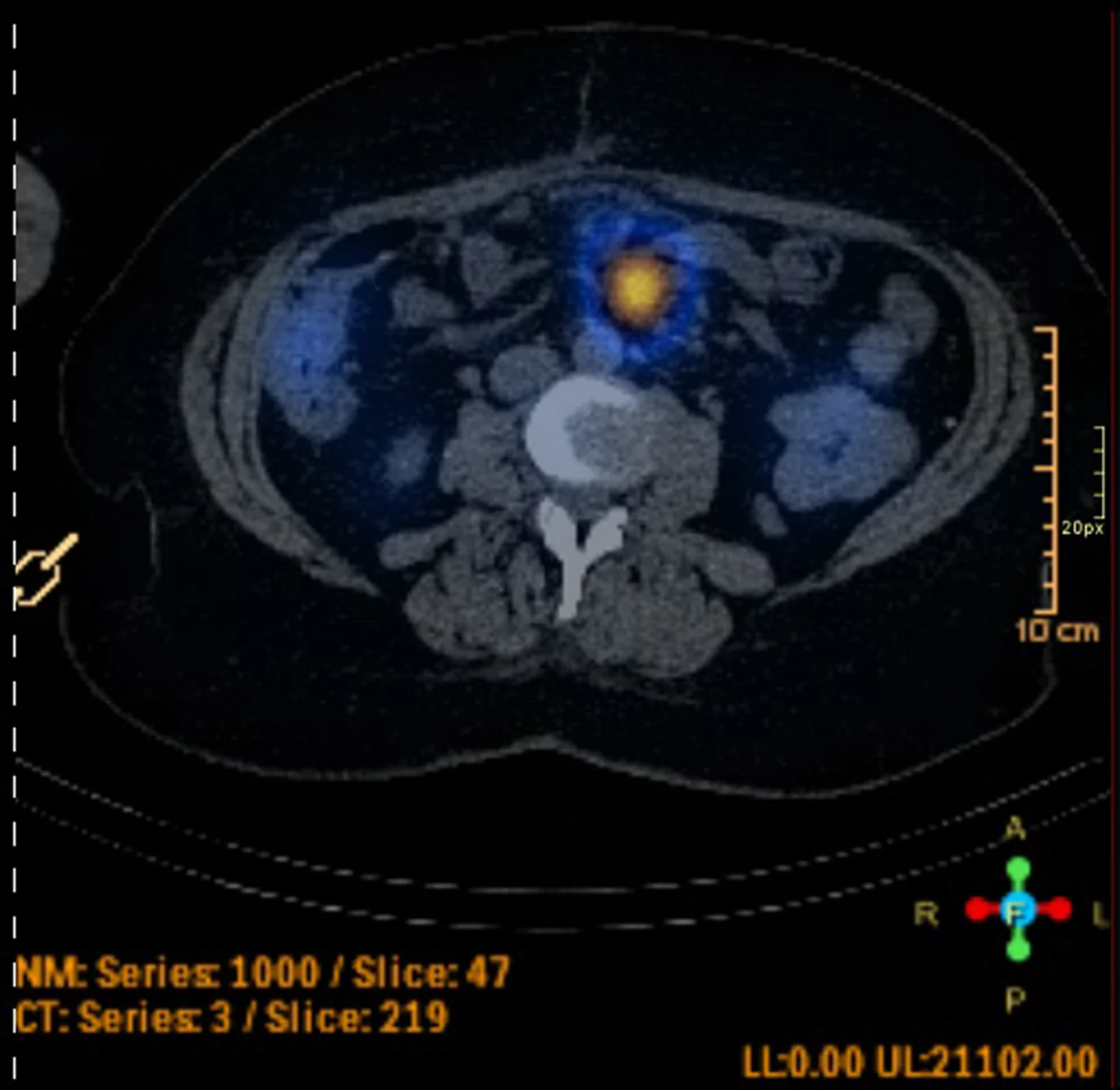
NM, octreotide SPECT (post 4# of PRRT).

No PRRT dose reductions, treatment delays, or treatment interruptions occurred during any of the 12 treatment cycles. Renal and hepatic function remained within normal limits throughout treatment. No clinically significant hematologic toxicity was observed during active PRRT.

By late 2023, as she approached the final PRRT cycle, progressive cytopenias were noted, initially involving erythroid and platelet lineages, followed by neutropenia. By early to mid-2024, she developed significant clinical symptoms including profound fatigue, recurrent infections, and worsening pancytopenia. This clinical deterioration was discordant with her sustained radiologic disease stability (Fig. 12, 13). Hematology consultation was obtained, and a bone marrow biopsy performed in September 2024 revealed therapy-related AML characterized by inv(3)(q21q26.2), monosomy 7, and a complex karyotype. Next-generation sequencing identified an activating KRAS mutation. Lanreotide therapy was discontinued at the time of leukemia diagnosis due to concern for exacerbation of anemia. There was no direct evidence establishing a causal relationship between lanreotide and the development of anemia. However, at the request of the hematology team, SSA therapy was discontinued as a precaution to mitigate any perceived risk of worsening anemia.

**Fig. 12.**
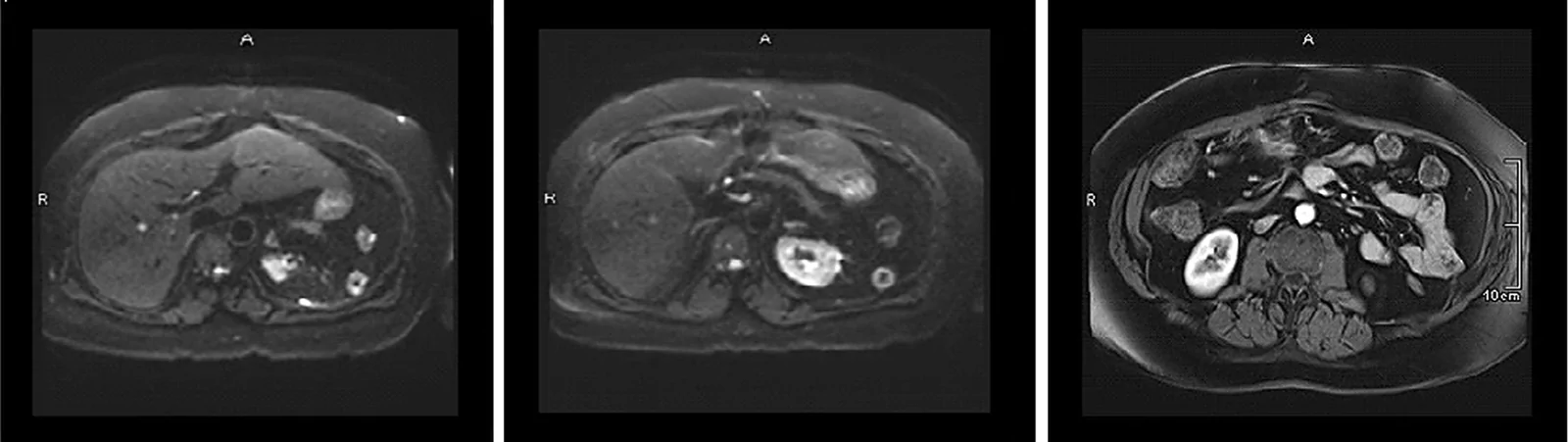
MRI, DWI (post 12# of PRRT).

**Fig. 13.**
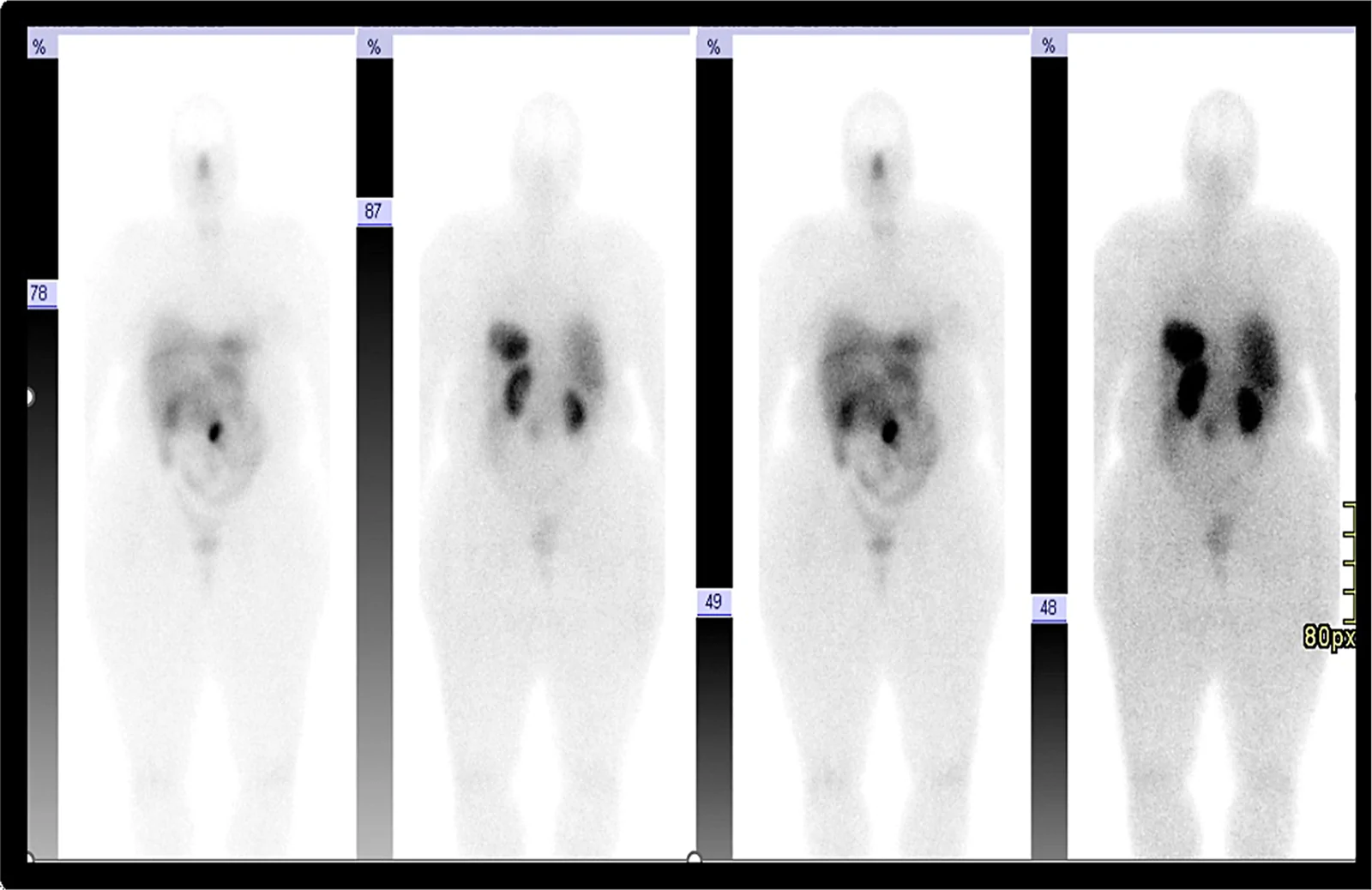
NM, octreotide SPECT (post 12# of PRRT).

She was admitted under the hematology service and received induction chemotherapy with FLAG-Ida (fludarabine, cytarabine, granulocyte colony-stimulating factor, and idarubicin) in October 2024; however, she failed to achieve a response. A repeat bone marrow biopsy in November 2024 confirmed refractory disease. Subsequently, lower-intensity therapy with azacitidine and venetoclax was initiated in November 2024 with palliative intent. Following the first cycle, she was readmitted with failure to thrive, refractory nausea and vomiting, and progressive vision loss. Brain MRI demonstrated abnormal gyriform enhancement in the right parieto-occipital and left parietal regions, as well as leptomeningeal enhancement. Despite extensive diagnostic evaluation, findings were most consistent with leukemic or paraneoplastic central nervous system involvement and did not respond to high-dose methylprednisolone.

Her clinical condition continued to deteriorate, marked by progressive encephalopathy, bilateral vision loss, fluctuating delirium, persistent pancytopenia, multiorgan dysfunction, and eventual organ failure. Despite aggressive supportive care, she died on January 13, 2025. The cause of death was attributed to refractory therapy-related AML with central nervous system involvement (see Tables 1, 2).

**Table 1.** Chronological course of patient’s clinical events (summarized)

Time	Clinical milestone	Key findings and management
2017	Initial diagnosis	Presented with abdominal pain, loose stools, and a palpable abdominal mass; biopsy confirmed a metastatic, well-differentiated grade 1 small-bowel NET (Ki-67 <2%), with elevated 5-HIAA and chromogranin A
2018	Cytoreductive surgery	Underwent small-bowel resection and hepatic wedge resection; the dominant mesenteric mass was unresectable and remained in situ because of extensive involvement of the superior mesenteric vessels
2018	SSA therapy	Octreotide LAR 30 mg every 4 weeks was initiated
September 2018	Disease progression	Radiologic progression during SSA therapy prompted enrollment in the provincial ^177^Lu-DOTATATE clinical trial
September 2018–November 2023	Extended ^177^Lu-DOTATATE treatment	Completed 12 cycles – 4 induction and 8 maintenance cycles – with a cumulative administered activity of 48.000 GBq; durable biochemical and radiologic disease control was achieved. No toxicity-related dose reductions, delays, or treatment interruptions occurred
December 2023	Early hematologic changes	Mild anemia was documented after completion of cycle 12 (Hb, 107 g/L), without concurrent renal or hepatic dysfunction
June 2024	Progressive cytopenias	Progressive pancytopenia developed: Hb, 108 g/L; ANC, 0.5 × 10^9^/L; and platelets, 116 × 10^9^/L
10 September 2024	Severe pancytopenia	Required her first blood transfusion after Hb decreased to 72 g/L and platelets to 9 × 10^9^/L; an urgent bone marrow examination was performed
23 September 2024	Therapy-related AML	Bone marrow examination demonstrated hypercellularity, trilineage dysplasia, and 48–50% blasts. Cytogenetic and molecular investigations identified inv(3)(q21q26.2)/MECOM rearrangement, monosomy 7, a complex karyotype, and a pathogenic KRAS mutation; FLT3 testing was negative
October–November 2024	Refractory AML	Received FLAG-Ida induction therapy; repeat bone marrow examination demonstrated refractory disease with 64% blasts. Treatment was transitioned to azacitidine plus venetoclax with palliative intent
13 January 2025	Outcome	Died from refractory therapy-related AML with central nervous system involvement and multiorgan dysfunction

5-HIAA, 5-hydroxyindoleacetic acid; AML, acute myeloid leukemia; ANC, absolute neutrophil count; CNS, central nervous system; Hb, hemoglobin; LAR, long-acting release.

**Table 2.** Administration of ^177^Lu-DOTATATE and serial hematologic monitoring of the patient

Cycle	Administration date	Activity, MBq	Activity, GBq	Cumulative activity, GBq	Hb, g/L	WBC, ×10^9^/L	ANC, ×10^9^/L	Platelets, ×10^9^/L	Renal and hepatic function
1	10 September 2018	5,301	5.301	5.301	108	6.7	4.0	291	Normal
2	21 November 2018	5,424	5.424	10.725	115	5.2	3.2	261	Normal
3	2 April 2019	5,731	5.731	16.456	117	4.4	3.3	246	Normal
4	3 July 2019	4,380	4.380	20.836	120	4.7	3.4	266	Normal
5	11 March 2020	3,892	3.892	24.728	123	4.7	3.0	276	Normal
6	9 September 2020	3,848	3.848	28.576	113	4.5	3.0	241	Normal
7	5 May 2021	3,339	3.339	31.915	126	4.5	3.3	266	Normal
8	3 November 2021	3,912	3.912	35.827	123	4.5	3.1	259	Normal
9	7 June 2022	3,792	3.792	39.619	117	4.1	2.8	265	Normal
10	23 November 2022	2,673	2.673	42.292	119	2.9	1.5	270	Normal
11	30 May 2023	2,779	2.779	45.071	119	4.0	2.7	262	Normal
12	28 November 2023	2,929	2.929	48.000	121	4.8	3.5	292	Normal

The hematologic values shown are those obtained closest to each treatment cycle. No toxicity-related dose reductions, treatment delays, or interruptions occurred. Renal and hepatic function remained normal throughout treatment, and clinically significant hematologic toxicity became apparent only after completion of cycle 12.

ANC, absolute neutrophil count; GBq, giga-becquerel; Hb, hemoglobin; MBq, megabecquerel; WBC, white blood cell count.

## Discussion

TR-MNs constitute a devastating late complication of cytotoxic therapy and radiation exposure and are uniformly associated with poor clinical outcomes [14]. Although PRRT-associated TR-MNs are infrequently reported, cumulative radiation exposure to the bone marrow represents a biologically plausible mechanism of leukemogenesis, particularly in patients without prior exposure to cytotoxic chemotherapy or external-beam radiation [15, 16].

Reported latency periods between PRRT exposure and the development of hematologic malignancies range from 15 to 84 months, consistent with the temporal course observed in the present case [17]. Several studies have suggested an increased risk of hematologic toxicity in patients with advanced age, impaired renal function, baseline cytopenias, high tumor burden, and high tumor uptake on octreotide scintigraphy; however, these associations remain inconsistent across studies [17]. Notably, bone marrow dosimetry has failed to reliably predict subsequent hematologic toxicity. These findings contrast with results from a Dutch cohort that specifically evaluated potential risk factors for hematologic toxicity following 177Lu therapy and reported no statistically significant predictors. The median latency to AML diagnosis has been reported to be approximately 39 months, shorter than that observed for therapy-related MDSs [18].

In a large retrospective analysis of 1,631 patients treated with PRRT, approximately 30 individuals developed TR-MNs, including 7 cases of AML. Outcomes were uniformly poor, with a median overall survival of approximately 13 months for TR-MNs overall, 15 months for MDS, and only 6 months for AML [18]. Despite multiple efforts to define predictive factors for PRRT-related hematologic toxicity, no validated or universally accepted risk stratification model currently exists. Although the absolute incidence of TR-MNs following PRRT remains low, the associated morbidity, aggressive disease biology, and markedly reduced survival underscore the clinical significance of this complication.

Globally, the standard ^177^Lu-DOTATATE regimen consists of four cycles, as established by the NETTER trials. NETTER-1 established four cycles of ^177^Lu-DOTATATE as an effective treatment for progressive midgut NETs, demonstrating substantial improvements in PFS and objective response rate compared with high-dose octreotide [7, 10]. NETTER-2 subsequently demonstrated improved PFS and response rates with first-line ^177^Lu-DOTATATE in patients with newly diagnosed grade 2–3 GEP-NETs [8]. Although hematologic toxicity was uncommon in both trials, long-term data indicate an estimated incidence of therapy-related MDS of 2–4% and acute leukemia of less than 1%. Reported risk factors include advanced age, bone metastases, extensive prior treatment, and exposure to alkylating agents such as temozolomide [11].

Evidence supporting additional maintenance cycles remains limited, and the safety of extending therapy to 12 cycles has not been established in large clinical trials. The administration of eight additional maintenance cycles following radiologic tumor stability needs to be further supported by phase III data and has not been systematically justified or reported in the literature. Consequently, the hematologic safety profile of prolonged PRRT exposure – particularly regimens involving 12 cycles – remains undefined. The absence of formally published safety data limits the ability to draw definitive conclusions and underscores an important knowledge gap regarding the clinical rationale for extending PRRT beyond four cycles. These observations highlight the need for further evaluation of optimal treatment duration, particularly with respect to balancing sustained disease control with the potential risk of long-term adverse outcomes.

To date, the only published study reporting outcomes following 12 cycles of PRRT is a Canadian phase II trial conducted at Western University in London, ON, which explored expanded use of ^177^Lu-DOTATATE beyond induction-only protocols [19]. This study employed individualized dosing and extended maintenance therapy, including non-midgut NETs, and reported a median PFS of 36.1 months. Notably, patients receiving PRRT as first-line systemic therapy following SSA failure demonstrated a median PFS of 48.9 months, among 47 PRRT-naïve patients. Promising efficacy was particularly observed in midgut NETs, with a median PFS of 47.7 months. However, several limitations restrict the generalizability of these findings, including the single-arm, non-randomized design and the inclusion of a heterogeneous NET population. Additionally, differential response rates were observed across tumor subtypes, with pancreatic NETs demonstrating higher partial response rates (69.2%) compared with GEP-NETs (5%), suggesting subtype-specific radiosensitivity. Variability in prior therapies, small sample size, and the absence of published long-term safety outcomes further limit comparability with pivotal randomized trials. Despite attempts at dose individualization based on renal function, hepatic function, and hematologic parameters, insufficient data exist to define the optimal number of PRRT cycles required for durable benefit. While this study demonstrates a PFS benefit associated with flexible dosing and extended PRRT in carefully selected patients, further research is needed to better characterize cumulative toxicity risks, confirm potential survival advantages, and establish comprehensive long-term safety data that may support wider adoption of extended PRRT regimens in clinical practice.

Emerging evidence implicates clonal hematopoiesis (CH) as a potential predictive biomarker for PRRT-related hematologic toxicity. Clonal hematopoiesis of indeterminate potential (CHIP) is defined by the presence of somatic mutations associated with hematologic malignancies in individuals without overt hematologic disease. Identification of CH prior to PRRT initiation may provide valuable prognostic insight and enable risk-adapted treatment strategies [20, 21]. In a recently published exploratory study involving 13 patients, 62% of individuals with CH at baseline harbored TP53, PPM1D, or CHEK2 mutations and subsequently developed varying degrees of pancytopenia following PRRT [22]. Although these findings are compelling, routine CH testing prior to PRRT has not yet been incorporated into standard clinical practice.

In a retrospective study of 807 patients with NETss treated with various PRRT regimens, Bodei et al. [23] reported MDS and acute leukemia in 2.35% and 1.1% of patients, respectively, confirming that TR-MNs are uncommon but clinically important late complications. Prolonged treatment duration and greater platelet toxicity were associated with MDS, while the mean interval from PRRT initiation to acute leukemia was approximately 4.7 years. Clinical and treatment-related factors explained only a minority of cases, and bone marrow dosimetry was not predictive, suggesting that individual biological or genetic susceptibility may contribute to leukemogenesis. These findings are particularly relevant to our patient, who developed high-risk AML after receiving 12 cycles of ^177^Lu-DOTATATE over 5 years without prior cytotoxic chemotherapy or external-beam radiotherapy, and support the need for cautious evaluation of extended treatment regimens and long-term hematologic surveillance.

To facilitate early detection of hematologic toxicity, current NCCN guidelines recommend serial complete blood count monitoring at approximately 1, 3, 6, and 12 months following PRRT. In the presence of cytopenias, intensified surveillance is advised, and persistent abnormalities should prompt timely referral to hematology [24, 25].

Importantly, this patient lacked recognized clinical risk factors for TR-MN development, including advanced age, renal impairment, baseline cytopenias, or prior exposure to cytotoxic chemotherapy, alkylating agents, or external-beam radiotherapy. This case therefore supports a plausible association between extended ^177^Lu-DOTATATE exposure and leukemogenic risk. Nevertheless, alternative explanations for AML development must be acknowledged, including pre-existing clonal hematopoiesis, an undetected germline predisposition, de novo AML, and environmental exposures, particularly given the absence of baseline CH/CHIP testing.

The AML observed in our patient was characterized by a constellation of adverse cytogenetic and molecular features, including inv (3) (q21q26.2), monosomy 7, complex karyotype, and a pathogenic KRAS mutation. This genomic profile defines a rare and exceptionally high-risk subtype of AML associated with intrinsic chemoresistance, rapid clinical progression, and dismal prognosis. Inversion 3 and monosomy 7 are independently linked to poor treatment response and inferior survival outcomes; their coexistence, compounded by a KRAS mutation, further intensifies the adverse risk category. This subtype is notoriously refractory to conventional induction regimens, frequently demonstrates early relapse, and is often accompanied by limited tolerance to cytotoxic therapy. Despite aggressive treatment approaches, overall survival in this subgroup remains exceedingly poor [26].

This case provides important insight into an uncommon but clinically significant hematologic complication following extended ^177^Lu-DOTATATE therapy. The findings highlight valuable opportunities to further strengthen the safe and effective use of PRRT by

1. defining hematologic risks associated with prolonged PRRT exposure;
2. establishing evidence-based criteria for PRRT maintenance therapy;
3. implementing structured, long-term hematologic surveillance protocols for PRRT-treated patients;
4. investigating predictive biomarkers, including clonal hematopoiesis, to enable risk-adapted patient selection and treatment strategies.

The scientific discussion of this case report was prepared and reported in accordance with the CARE (CAse REport) guidelines. The completed CARE checklist is provided as online supplementary material.

## Conclusions

This case offers important insight into a rare and clinically significant occurrence of high-risk therapy-related AML following prolonged exposure to ^177^Lu-DOTATATE in a patient without prior cytotoxic chemotherapy, alkylating agents, external-beam radiation, or identifiable predisposing risk factors. Notably, the patient’s NET remained biochemically and radiologically stable throughout extended treatment with ^177^Lu-DOTATATE, illustrating a unique clinical scenario in which durable tumor control coincided with the emergence of an unexpected hematologic malignancy. These findings raise concern that cumulative marrow irradiation from ^177^Lu-DOTATATE may have contributed to leukemogenesis in this case. Alternative explanations for AML development must also be acknowledged, including pre-existing clonal hematopoiesis, an undetected germline predisposition, de novo AML, and environmental exposures, particularly given the absence of baseline clonal hematopoiesis (CH/CHIP) testing.

This report provides important insight into the safety considerations of PRRT regimens that extend beyond the currently approved four-cycle standard. In the absence of prospective phase III evidence supporting maintenance or extended-cycle approaches, further evaluation is needed to better define the benefit-risk profile of prolonged PRRT exposure. These findings emphasize the value of continued vigilance, including structured and long-term hematologic monitoring, particularly for patients receiving repeated PRRT over extended periods.

Furthermore, this case highlights critical gaps in current clinical knowledge, including the lack of validated risk stratification tools, uncertainty surrounding cumulative marrow dose thresholds, and the absence of evidence-based criteria guiding ^177^Lu-DOTATATE duration. Future research must focus on defining safe upper limits of ^177^Lu-DOTATATE exposure, establishing standardized monitoring protocols, and identifying predictive biomarkers, such as clonal hematopoiesis, to enable individualized, risk-adapted treatment decisions.

Ultimately, while ^177^Lu-DOTATATE remains a highly effective and transformative therapy for patients with advanced GEP-NETs, this case underscores the necessity of balancing long-term disease control against the potential for irreversible and life-threatening toxicity. Careful patient selection, adherence to evidence-based treatment duration, and continued investigation into long-term safety are essential to optimizing outcomes and preventing avoidable harm.

## Statement of Ethics

This study was performed in accordance with the Declaration of Helsinki. Ethical approval was not received for this human study because ethical approval was not required for this study in accordance with local and national guidelines as the patient data and images were fully anonymized and de-identified prior to analysis. Written informed consent was obtained from the patient husband (who served as the legally authorized representative) for publication of the details of their medical case and any accompanying images.

## Conflict of Interest Statement

The authors declare no conflicts of interest.

## Funding Sources

This research received no external funding.

## Author Contributions

A.A. contributed to manuscript finalization, clinical data curation, original draft preparation, supervision, project administration, and critical revision of the manuscript. S.H. contributed to study conceptualization, clinical data acquisition, investigation, and preparation of the original manuscript draft. M.A.-B. contributed to manuscript formatting, finalization, and online publication.

## Data Availability

The data supporting the findings of this study are not publicly available due to privacy and confidentiality considerations but may be made available from the corresponding author upon reasonable request. Further inquiries may be directed to the corresponding author.
